# MicroRNA 193b-3p as a predictive biomarker of chronic kidney disease in patients undergoing radical nephrectomy for renal cell carcinoma

**DOI:** 10.1038/bjc.2016.329

**Published:** 2016-11-01

**Authors:** Francesco Trevisani, Michele Ghidini, Alessandro Larcher, Andrea Lampis, Hazel Lote, Paolo Manunta, Maria Teresa Sciarrone Alibrandi, Laura Zagato, Lorena Citterio, Giacomo Dell'Antonio, Cristina Carenzi, Giovambattista Capasso, Massimo Rugge, Paolo Rigotti, Roberto Bertini, Luciano Cascione, Alberto Briganti, Andrea Salonia, Fabio Benigni, Chiara Braconi, Matteo Fassan, Jens Claus Hahne, Francesco Montorsi, Nicola Valeri

**Affiliations:** 1 Division of Molecular Pathology, The Institute of Cancer Research, London, UK; 2 Department of Urology, San Raffaele Scientific Institute, Milan, Italy; 3 Division of Oncology/Unit of Urology; IRCCS Ospedale San Raffaele, Milan, Italy; 4 Division of Nephrology and Dialysis, IRCCS San Raffaele Scientific Institute, Milan, Italy; 5 Genomics of Renal Disease and Hypertension Unit, IRCCS San Raffaele Scientific Institute, Milan, Italy; 6 Department of Pathology, San Raffaele Scientific Institute, Milan, Italy; 7 Department of Nephrology, Second University of Naples, Naples, Italy; 8 Department of Medicine, Surgical Pathology & Cytopathology Unit, University of Padua, Padua, Italy; 9 Department of Surgical, Oncological and Gastroenterological Sciences, Kidney and Pancreas Transplantation Unit, University of Padua, Padua, Italy; 10 Bioinformatics Core Unit, Institute of Oncology Research, Bellinzona, Switzerland; 11 Division of Cancer Therapeutics, The Institute of Cancer Research, London, UK; 12 Department of Medicine, The Royal Marsden NHS Trust, London, UK

**Keywords:** kidney cancer, radical nephrectomy, chronic kidney disease, clear-cell renal carcinoma, microRNAs, biomarkers

## Abstract

**Background:**

A significant proportion of patients undergoing radical nephrectomy (RN) for clear-cell renal cell carcinoma (RCC) develop chronic kidney disease (CKD) within a few years following surgery. Chronic kidney disease has important health, social and economic impact and no predictive biomarkers are currently available. MicroRNAs (miRs) are small non-coding RNAs implicated in several pathological processes.

**Methods:**

Primary objective of our study was to define miRs whose deregulation is predictive of CKD in patients treated with RN. Ribonucleic acid from formalin-fixed paraffin embedded renal parenchyma (cortex and medulla isolated separately) situated >3 cm from the matching RCC was tested for miR expression using nCounter NanoString technology in 71 consecutive patients treated with RN for RCC. Validation was performed by RT–PCR and *in situ* hybridisation. End point was post-RN CKD measured 12 months post-operatively. Multivariable logistic regression and decision curve analysis were used to test the statistical and clinical impact of predictors of CKD.

**Results:**

The overexpression of miR-193b-3p was associated with high risk of developing CKD in patients undergoing RN for RCC and emerged as an independent predictor of CKD. The addition of miR-193b-3p to a predictive model based on clinical variables (including sex and estimated glomerular filtration rate) increased the sensitivity of the predictive model from 81 to 88%. *In situ* hybridisation showed that miR-193b-3p overexpression was associated with tubule-interstitial inflammation and fibrosis in patients with no clinical or biochemical evidence of pre-RN nephropathy.

**Conclusions:**

miR-193b-3p might represent a useful biomarker to tailor and implement surveillance strategies for patients at high risk of developing CKD following RN.

Renal clear-cell carcinoma (RCC) is the most common type of kidney tumour in adults (85%) and accounts for 3% of all malignancies (Ferlay *et al*, 2013; Siegel *et al*, 2016). Surgery represents the gold standard for the treatment of clinically localised RCC (Ljungberg *et al*, 2015). Although oncological outcomes (in tumours measuring <4 cm) are similar in radical nephrectomy (RN) and in nephron sparing surgery (NSS), the incidence of long-term morbidity due to the development of chronic kidney disease (CKD) is higher in the case of RN due to the loss of nephron mass (Capitanio *et al*, 2016). Chronic kidney disease affects quality of life and life expectancy and has important health-economic implications being associated with increased cardiovascular risk, metabolic syndromes and end-stage kidney disease (Nashar and Egan, 2014).

Even though several mechanisms for the decay in renal function following RN have been hypothesised, no predictive markers are currently available to inform clinicians of the risk of developing CKD and to tailor surveillance and secondary prevention programs (Capitanio *et al*, 2016).

MicroRNAs (miRs) are short (19–24 nucleotides) non-protein coding RNAs fine tuning cell homeostasis by controlling gene expression at post-transcriptional level (Fabbri *et al*, 2009; Winter *et al*, 2009). MiRs are implicated in renal physiology regulating ion transport, electrolytes and acid–base equilibrium, as well as blood pressure (Elvira-Matelot *et al*, 2010; Mladinov *et al*, 2013; Trionfini *et al*, 2015). MiRs deregulation is common to many cancers as well as infectious, cardiovascular, metabolic and kidney diseases and it can be exploited for screening, diagnosis and treatment (Catto *et al*, 2011; Mendell and Olson, 2012; Fasanaro *et al*, 2015; Trionfini *et al*, 2015).

In the current study, we aimed to test miR deregulation in ‘normal' kidney tissue in patients with and without deterioration in renal function after RN for RCC, in order to define predictive biomarkers of CKD.

## Materials and methods

### Study population

One-hundred and five patients treated with RN for RCC at San Raffaele Hospital (Milan, Italy) between 2008 and 2013 were eligible for the study. The project has been approved by the relevant Ethic Committee (approval 2007/29082007/V3) and informed consent is available for each patient. The inclusion criteria of the study were: (a) age >18 and <80 years; (b) clear-cell carcinoma histology; (c) resectable RCC and absence of metastatic within the initial 12 months from diagnosis; (d) no history of other malignancies; (e) normal renal function prior to RN, estimated glomerular fraction rate (eGFR)>60 ml min^-1^ (CKD-EPI formula 2009 (Eknoyan *et al*, 2013)), serum creatinine <1.1 mg dl^-1^ and no urinary abnormalities (i.e., proteinuria); (f) absence of primitive, secondary, hereditary or acquired glomerulopathies; (g) absence of kidney stone disease, myeloproliferative disorders and autoimmune diseases (i.e., vasculitis and systemic infections); (h) absence of chronic nephrotoxic drug therapy (e.g., lithium, NSAID). All patients underwent postoperative review 1 month after RN and 6-month follow-up with physical examination, lab test and CT scan thereafter.

Twelve renal biopsies (cortex only) from kidney donors (Remuzzi score ⩽4 (Remuzzi *et al*, 1999)) were collected from the archives of the Surgical Pathology Unit at Padua University (Padua, Italy) and used as controls (Ethic Committee approval #: 0011418–02/03/2015).

### Pathology review

Haematoxylin and eosin-stained slides from 100 formalin-fixed paraffin-embedded tissues were reviewed by a pathologist. Only samples >3 cm distance from RCC were considered. Renal cortex and medulla were manually micro-dissected in order to separate the glomerular region from the loop of Henle and collecting ducts. Twenty cases were excluded due to inadequate representation of the cortex region (<10 glomeruli on × 10 field of view), because this might have impaired the yield of RNA from the glomeruli.

Total RNA extraction was performed using Ambion Recover All Isolation Kit (Life Technologies, Carlsbad, CA, USA), according to the manufacturer's instructions. The purity and quality of extracted RNA was determined by Bio-Analyser (Agilent Technologies, Santa Clara, CA, USA). Nine samples were discarded due to poor RNA purity/quality.

### NanoString nCounter and bioinformatics analysis

MiR expression was analysed using the nCounter Human v2 miR Expression Assay kit (NanoString, Seattle, WA, USA) as we previously described (Valeri *et al*, 2014). This assay detects 800 endogenous miRs, 5 housekeeping transcripts plus 6 positive and 6 negative controls. About 150 ng of each total RNA sample was used as input into the nCounter Human miR sample preparation. Hybridisation was conducted for 16 h at 65 °C. Subsequently, the strip tubes were placed into the nCounter Prep Station for automated sample purification and subsequent reporter capture. Each sample was scanned for 555 fields of view on the nCounter Digital Analyzer (NanoString). Data were extracted using the nCounter RCC Collector (NanoString). Two samples, one medulla from a patient with normal kidney function (NKF) and one cortex from a healthy kidney donor, failed quality control and were excluded from further analysis.

Raw data, which are proportional to copy number, were log-transformed and normalised by the quantile method after application of a manufacturer-supplied correction factor for several miR (Cascione *et al*, 2013). Data were filtered to exclude relatively invariant features (IQR=0.5) and features below the detection threshold (defined for each sample by a cutoff corresponding to ∼2 × s.d. of negative control probes plus the mean of them) in at least half of the samples. After the pre-processing steps described above, we plotted the relative differences in transcriptional profile between the samples using multidimensional scaling plot. Using R/Bioconductor and the filtered data set, significance analysis of microarrays was employed with a contrast matrix for the comparisons (Cascione *et al*, 2013). *P*-values were used to rank miRs of interest, and correction for multiple comparisons was performed using the Benjamini–Hocheberg method (Benjamini and Hochberg, 1995). Raw data that were above background, as well as the corresponding quantile-normalised data, were imported into MultiExperiment Viewer (http://mev.tm4.org/) for visual inspection.

Heatmaps were generated using the Euclidean distance as metric distance and a single clustering as linkage criterion. Only miRs with a *t*-test *P*-value <0.1 were included in the heatmaps. The colour red indicates strong expression of a miR, whereas a blue point mirrors a reduced level of a determined miR.

MicroRNA expression data have been submitted to GEO under accession number GSE80247.

### RT–PCR analysis

Expression of four of the most importantly dysregulated miRs detected using NanoString nCounter Analysis (hsa-miR-193b-3p, hsa-miR-365a-3p, hsa-miR-363b-3p and hsa-miR-139b-5p) was investigated by RT–PCR using Taqman assays (Life Technologies). Comparative RT–PCR was run in triplicate, including no template controls and endogenous control gene *RNU48*. The fold difference for each sample was obtained using the ΔCT method.

### miR-193b *in situ* hybridisation analysis

Locked nucleic acid (LNA) probes with complementarity to miR-193b-3p were labelled with 5′-biotin and synthesised using Exiqon (Vedbaek, Denmark). Tissue sections were digested with ISH protease 1 (Ventana Medical Systems, Milan, Italy) and ISH was performed as we previously described (Nuovo *et al*, 2012). Positive (U6; Exiqon) and negative scrambled LNA probes (Exiqon) were used as controls. Only cytoplasmic miR staining was retained for scoring purposes.

### Statistical analyses

The end point of the study was the rate of *de novo* post-RN CKD defined as stage 3a-3b-4 calculated 12 months after surgery (Eknoyan *et al*, 2013).

First, the Mann–Whitney test was used to compare the statistical significance of differences in the distribution of each miR of interest according to CKD. Second, multivariable logistic regression analysis (MVA) was used to construct a baseline model predicting the CKD relying on clinical predictors only. Such predictors were factors suggestive for increased risk of post-surgical CKD, and consisted of age, gender, baseline eGFR, clinical tumour size (defined as the largest tumour dimension at preoperative imaging) and presence of diabetes. Third, each miR of interest was independently added to the baseline model, and the concordance index of each miR-inclusive model was compared with that of the baseline model. Fourth, the net benefit of the baseline model and of each miR-inclusive model was assessed using decision curve analysis (Vickers and Elkin, 2006).

## Results

### Baseline clinical characteristics and measure of clinical outcome

**Figure 1:**
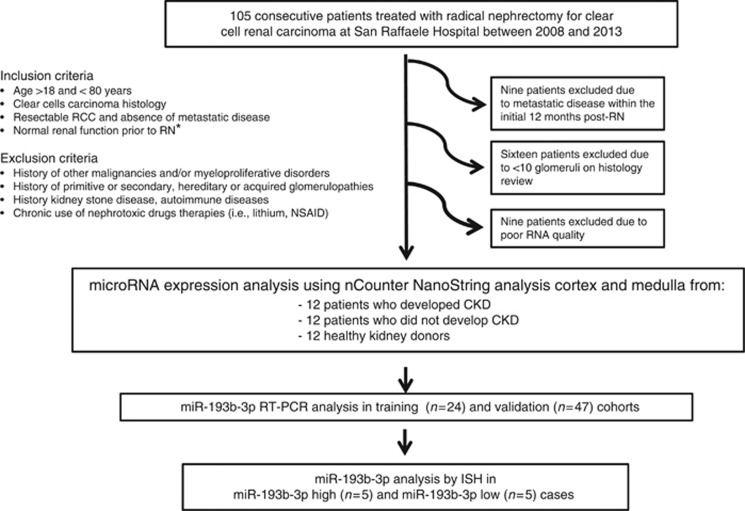
**Schematic overview of the study.** *The eGFR>60 ml min^-1^ based on CKD-EPI formula 2009. CKD, chronic kidney disease; eGFR, estimated glomerular fraction rate; ISH, *in situ* hybridisation; RN, radical nephrectomy.

Of the originally collected one-hundred and five patients with histologically proven, RCC treated with curative RN, 71 patients were included in our study for clinical and molecular predictors of CKD (34 patients were excluded from the analysis and details are shown in Figure 1). Patient characteristics are summarised in Table 1.

Fourteen (20%) patients suffered from type II diabetes control by oral hypoglycaemic agents at the time of diagnosis; no patients had type I diabetes (Table 1). Histological review of resection specimen ruled out signs of pre-existing nephropathy in all these patients. Similarly, none of the patients showed biochemical signs of CKD with normal renal function in the absence of proteinuria.

Physiological fluctuations in the eGFR are common in the post-RN setting, relate to compensatory nephron hyperfiltration in the contralateral kidney and, usually, occur within the initial 6 months from the operation. Given that several lines of evidence suggest that a reduction in eGFR at 12 months from RN is associated with increased risk of CKD (Westland *et al*, 2014), our end point measurement was performed 12 months post RN.

Thirty-eight (54%) patients experienced a reduction in eGFR, developing a mild to severe CKD 12 months post RN. (Supplementary Figure S1A and 1B).

### Identification of microRNAs associated with CKD in the training cohort

In order to define miRs discriminating patients who developed CKD at 12 months post RN and patients with NKF, we performed miR expression analysis in a randomly selected training cohort of 12 CKD and 12 NKF patients. As CKD can be related to different abnormalities affecting the nephron, the cortex and medulla were dissected and tested for miR expression separately.

Nine miRs were upregulated and one was downregulated >1.5-fold (*P*-value ⩽0.05) in the comparison between CKD and NKF patients (irrespective of the anatomic location) (Supplementary Table S1).

**Figure 2:**
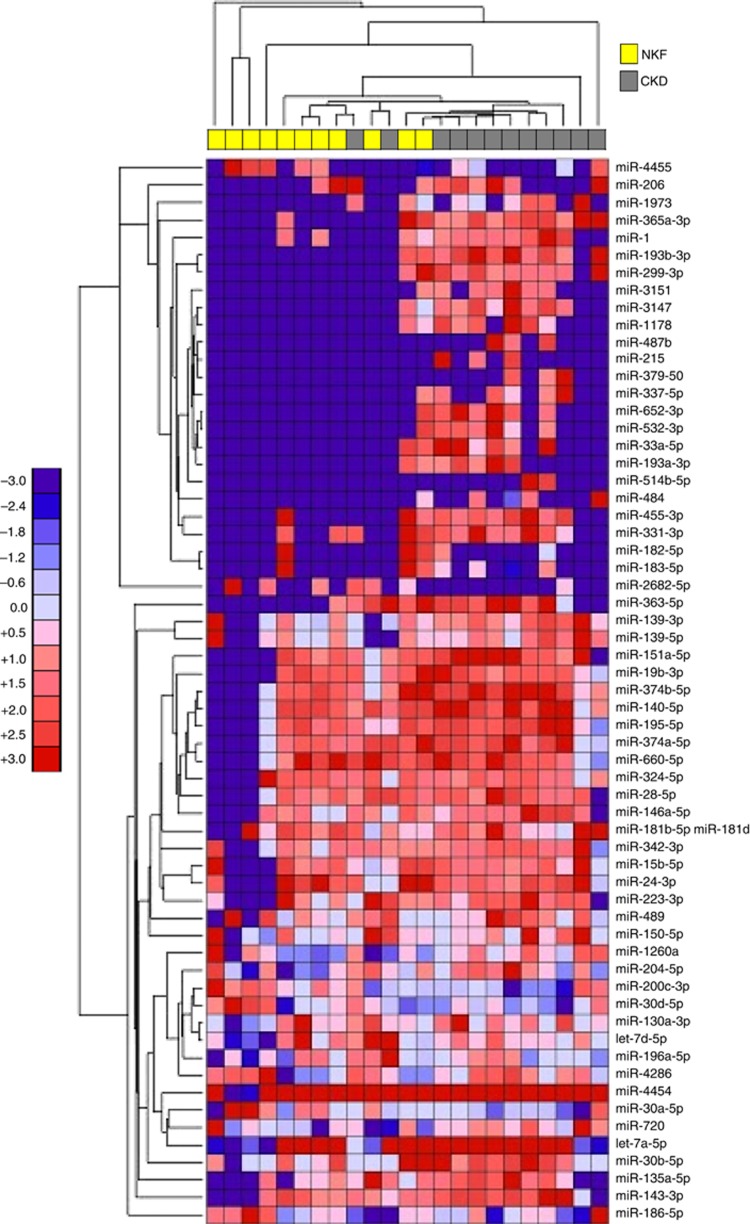
**Heatmap showing miR expression in medulla of patients with normal kidney function (NKF) compared with patients developing chronic kidney disease (CKD) 12 months post nephrectomy for RCC.** The miRs with a *P*-value ⩽0.1 are shown.

When the analysis compared the medulla of CKD and NKF patients, ten miRs were found to be overexpressed more than 1.5-fold in CKD patients. No miR showed statistically significant downregulation >1.5-fold (Figure 2 and Supplementary Table S2). Conversely, when the analysis focused on the cortex, two miRs were found to be upregulated and one miR was downregulated >1.5-fold in CKD patients (Supplementary Figure S2 and Supplementary Table S3).

### Shortlisting and validation of miRs found to be deregulated in CKD *vs* NKF patients

For the validation of miRs associated with the development of CKD, we focused on miRs upregulated in the comparison between medulla of CKD *vs* NKF patients based on the following observations: (1) CKD is largely due to early tubular dysfunction, which promotes increased sodium reabsorption and glomerular hyperfiltration (Capitanio *et al*, 2016); (2) the comparison between medulla of CKD *vs* NKF provided more significantly deregulated miRs than the comparison between cortex of the same patients; (3) overexpressed rather than silenced miRs might be easier to detect in tissues and biological fluids.

Expression of the four miRs (miR-193b-3p, miR-365a-3p, miR-363b-3p and miR-139b-5p) deregulated in the medulla of CKD *vs* NKF patients detected in the training cohort by nCounter was confirmed in the same cohort (*n*=23) and validated in the remaining patients (*n*=47) by RT–PCR. All four miRs were confirmed as significantly upregulated in the cortex of CKD patients (Supplementary Table S4).

### Clinical and molecular predictors of CKD

**Figure 3:**
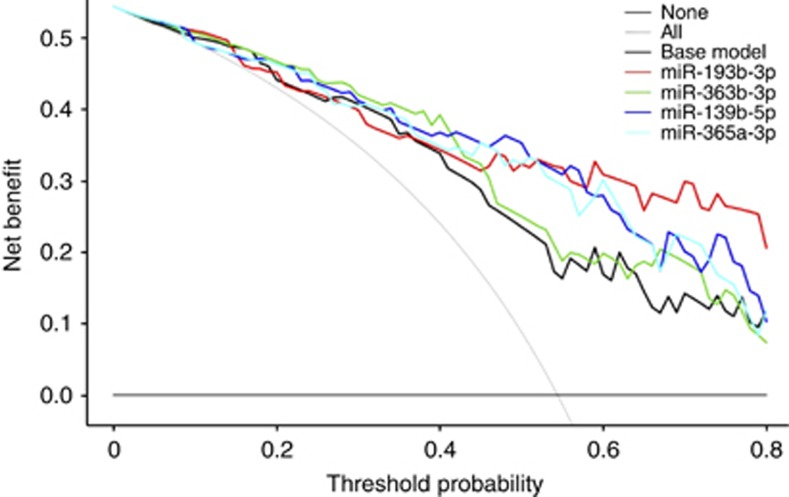
**Decision curve analysis plot comparing performance of a predictive score including clinical variables (base model) alone or in combination with specific miRs.**

In order to define clinical markers of CKD we performed a MVA, which showed that gender (odds ratio (OR) 6.07; *P*=0.02) and preoperative eGFR (OR 0.93; *P*=0.02) are independent predictors of CKD (base model (Table 2)). The area under the curve (AUC) of the base model was 81%. When each miR was included in the base model, each miR emerged as an independent predictor of CKD after adjustment for clinical variables. Specifically, miR-193b-3p (OR 2.28; *P*=0.002), miR-363b-3p, (OR 6.28; *P*=0.04), miR-139b-5p (OR 5.86; *P*=0.01) and miR-365a-3p (OR 2.18; *P*=0.01) were all associated with an increased risk of CKD. Moreover, after the inclusion of miR-193b-3p, miR363b-3p, miR139b-5p and miR365a-3p, the AUC of the base model increased to 88%, 83%, 86% and 86%, respectively (Table 2). At decision curve analyses (DCA), the inclusion of each miR into a baseline model predicting CKD using only clinical variables yielded a higher net benefit (Figure 3). In order to test whether the inclusion of multiple miRs to the basic model would yield higher predictive value, we selected model 1 (Table 2) as our base model (clinical-pathological variables+miR-193b-3p) and we added miR-363b-3p, miR-139b-5p and miR-365a-3p expression individually. None of these miRs increased the clinical and/or statistical power of the predictive model including miR-193b-3p alone (all *P*>0.05). Moreover, the inclusion of miR-363b, miR-139b and miR-365a in such model was associated with an AUC of 0.88, 0.89 and 0.89, respectively, which do not differ from the AUC of our original model 1 including clinical-pathological parameters+miR-193b expression (0.88). On the basis of these observations, we focused our attention on miR-193b-3p alone for further analysis.

### MiR-193b-3p tissue of origin

MiR expression is organ and tissue dependent (Lim *et al*, 2005). In order to validate the observations gathered from nCounter and RT–PCR data and provide insights in the role of these miRs in CKD pathogenesis, we performed miR-193b-3p ISH analysis in medulla and cortex tissues in samples characterised by high and low miR-193b-3p expression at nCounter and RT–PCR analysis in patients with high preoperative eGFR.

**Figure 4:**
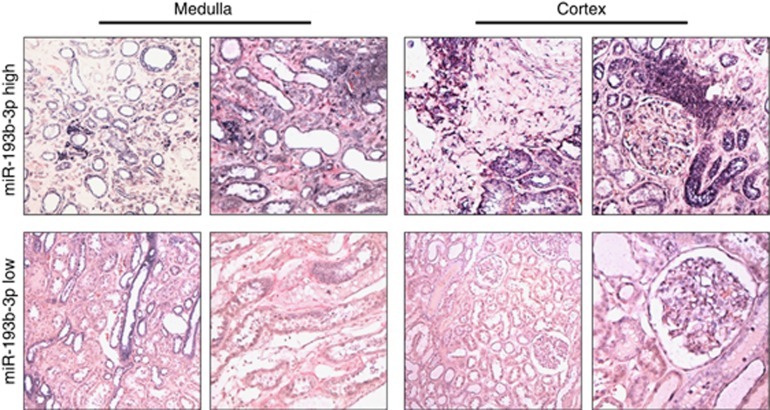
**MiR-193b-3p *in situ* hybridisation in ‘normal' parenchyma adjacent to RCC.** *In situ* hybridisation was conducted in samples characterised by high (*n*=5) and low (*n*=5) miR-193b-3p expression at nCounter and RT–PCR analysis in patients with high preoperative eGFR. The expression of miR was detectable as a grainy blue cytoplasmic staining. In cases with low miR-193b expression, only faint miR-193b staining was observed in the collector ducts in the medulla, and in distal convoluted tubule and in glomerular endothelial cells in the cortex. High-miR-193b cases were characterised by a moderate to strong miR expression in the inflammatory infiltrate and fibroblasts present in both renal compartments. A significant miR-193b overexpression was observed in atrophic and swollen tubules and ducts within the medulla.

In cases with low miR-193b-3p expression, faint miR-193b-3p staining was observed in the collector ducts (medulla), distal convoluted tubule and glomerular endothelial cells (cortex). High-miR-193b-3p cases were characterised by strong miR expression in the inflammatory infiltrate and in fibroblasts present in both renal regions. Moreover, in keeping with the nCounter expression data, significant miR-193b-3p overexpression was observed in atrophic and swollen tubules and in ducts within the medulla (Figure 4).

Cancer can cause re-wiring of miR and gene expression in adjacent surrounding tissues (Volinia *et al*, 2010). In order to test whether miR-193b-3p expression changes in renal parenchyma were caused by the cancer, we compared global miR expression in patients who underwent RN for RCC with healthy kidney donors. Even though a global miR deregulation was observed in ‘normal' tissues surrounding cancer compared with healthy donors, miR-193b-3p was not among the deregulated miRs (Supplementary Figures S3 and S4 and Supplementary Table S5). To support this observation, we also analysed miR-193b-3p expression in RCC patients in The Cancer Genome Atlas (TCGA (Cancer Genome Atlas Research N, 2013)) data set for whom cancer and matching normal miR-Seq data were available. Comparison of cancer and adjacent tissues confirmed that miR-193b-3p is overexpressed by 40% (*P*<0.0001) in normal tissues adjacent to the cancer (Supplementary Figure S5).

## Discussion

Three quarters of patients affected by stage I–II RCC will survive more than 5 years from the initial diagnosis (Karakiewicz *et al*, 2007). Although several efforts to improve oncological outcome in early and metastatic RCC patients are ongoing, little has been done to reduce comorbidities and improve quality of life and outcome in cancer survivors.

The incidence of CKD is increasing worldwide and the association between RN and increased risk of CKD is well documented (Mariusdottir *et al*, 2013), highlighting the unmet need for predictive biomarkers that might enable us to tailor patients' surveillance and treatment.

MiR deregulation has been studied in RCC and diabetes-related kidney disorders (Catto *et al*, 2011; Trionfini *et al*, 2015), but no study has linked miR deregulation with the risk of developing CKD in RN patients.

Our study led to two main conclusions: (1) the use of miR-193b-3p improves the predictive value of clinical nomograms in estimating the risk of CKD; (2) miR-193b-3p upregulation is associated with morphological features of inflammation, fibrosis and atrophy in patients with no preoperative clinical or biochemical evidence of kidney disease.

Our data rely on the retrospective analysis of a limited cohort of patients, thus are not intended to change clinical practice. However, while prospective validation studies are ongoing, questions and avenues of future research have arisen.

MiR-193b-3p has been previously characterised as a regulator of transcription factors such as MYB (Mets *et al*, 2015), downstream effector of MAPK (Ikeda *et al*, 2012) pathway and regulator of cell steaminess (Haetscher *et al*, 2015) in different tumour types including pancreatic cancers and leukaemia. Similarly, miR-193b-3p dysregulation has been detected in rheumatic disorders (Iwamoto *et al*, 2016), pre-eclampsia (Xu *et al*, 2014), as well as inflammation in white adipose tissues (Arner *et al*, 2012) through the regulation of inflammatory chemokines.

In our cohort, miR-193b-3p overexpression is associated with inflammation and atrophy and can be detected in tubular, ductal and inflammatory cells suggesting crosstalk between different compartments in promoting inflammation and fibrosis.

A reasonable question is whether the cancer is promoting inflammatory changes and miR-193b-3p overexpression. It is worth noticing that miR-193b-3p upregulation was independent of tumour and/or kidney size and no evidence of compression from the cancer was observed on histological review, excluding an indirect effect of malignancy in promoting an inflammatory/hypoxic reaction in the normal counterpart. Exosome transfer of miR has been widely studied in the interaction between tumour and its surrounding tissues (Neviani and Fabbri, 2015), thus it is legitimate to speculate that overexpression of miR-193b-3p in cancer could have driven the phenotype we observed. Some observations, however, argue against this theory, as the analysis of an independent cohort of RCC cases and matching controls included in the TCGA data set revealed a downregulation of miR-193b-3p in cancer compared with normal tissues; an observation that fits with several reports suggesting a tumour suppressor role for miR-193b-3p (Gastaldi *et al*, 2014; Mets *et al*, 2015). Further evidence supporting this hypothesis is represented by the comparison of RCC-matched normal tissues *vs* healthy kidney donors. Even though a general re-wiring of miR expression, possibly due to the cancer microenvironment, was observed, miR-193b-3p was not found to be deregulated in this comparison, suggesting that its upregulation might be independent from cancer.

A MVA for CKD suggested that basal eGFR was an independent predictor of CKD; however, even patients with high basal eGFR experienced a pathological reduction in glomerular filtration. As suggested by ISH analysis these patients showed early morphological signs of tubular-glomerular sclerosis associated with miR-193b-3p upregulation providing an explanation for the improved predictive value of the DCA when both parameters were included.

Similar to our observations, miR-193b-3p has been found to be upregulated in tissues and urine in patients with kidney interstitial fibrosis, tubular atrophy and acute kidney rejection, suggesting that this miR might be involved in pro-inflammatory feedback involving the interstitial compartment (Wilflingseder *et al*, 2013; Maluf *et al*, 2014).

The observation that miR-193b-3p upregulation appears independent from the cancer microenvironment and common to other inflammatory tubular-interstitial nephropathies led us to speculate that it might be indicative of a pre-existing, clinically and biochemically undetectable, disease potentially also affecting the contralateral kidney. In this scenario, the acute loss of nephron mass caused by nephrectomy could trigger hyperfiltration mechanisms, which in the presence of tubular atrophy and fibrosis in the remaining kidney could irreversibly compromise the tubular-glomerular feedback. Under these circumstances, afferent arteriolar vasodilatation, increased renal perfusion and stress for the glomeruli might translate into CKD within 12 months from the operation.

An interesting consideration is whether miR-193b-3p might be combined with other miRs to form a signature and whether this would be more accurate in defining the prognosis of RN patients than miR-193b-3p alone. We tested this hypothesis and we showed that including multiple miRs in the predictive model does not increase the performance of the model. Two potential explanations might justify this observation: (a) there is collinearity in miR-193b-3p and miR-363b-3p, miR-139b-5p and miR-365a-3p expression and as such there is no added predictive value in incorporating them together; (b) the number of events (patients with eGFR derangement at 12 months post RN) in our study is too limited to pick up a difference in predictive value.

Although the identification of miR-193b-3p targets is beyond the scope of our work, it is worth mentioning that several key features of CKD, such as inflammatory cell infiltration, tubular cell atrophy, mesangial cell hypertrophy and podocyte apoptosis, are linked to transforming growth factor-β (TGF-β) pathway activation (Trionfini *et al*, 2015). Interestingly miR-193b-3p has been shown to target TGF-β2- and TGFBR3 3′-untranslated regions suggesting a potential modulation of the pathway (Thiery, 2003; Hou *et al*, 2015; Zhou *et al*, 2016).

Our data suggest that integrating miR-193b-3p expression with clinical variables leads to a highly sensitive predictive model for the determination of risk of comorbidities in patients undergoing RN for RCC. Validation of our findings in prospective cohorts of patients receiving RN and NSS for kidney cancer is currently ongoing. Matched analysis of tissues, plasma and urine will test the robustness of this marker and its applicability to clinical practice.

## Figures and Tables

**Table 1:** Descriptive characteristics of 71 patients treated with radical nephrectomy for kidney cancer

**Variable**	**Overall**
Age, years	
Median	65
IQR	55–71
Gender	
Male	50 (70.4)
Female	21 (30.6)
Preoperative eGFR, ml min^-1^	
Median	90
IQR	78–97
Tumour size, mm	
Median	65
IQR	53–80
Kidney dimension, mm	
Median	125
IQR	110–145
Diabetes	
No	57 (80.2)
Yes	14 (20.8)

Abbreviations: eGFR=estimated glomerular fraction rate; IQR=interquartile range.

Data are presented as frequencies and percentages (numbers in brackets) unless otherwise specified.

**Table 2:** Logistic regression analysis predicting development of postoperative chronic kidney disease in 71 patients treated with radical nephrectomy for kidney cancer

	**Base model**		**Model 1–miR193b**		**Model 2–miR363b**		**Model 3–miR139b**		**Model 4–miR365a**	
**Predictors**	**OR (95% CI)**	***P*****-value**	**OR (95% CI)**	***P*****-value**	**OR (95% CI)**	***P*****-value**	**OR (95% CI)**	***P*****-value**	**OR (95% CI)**	***P*****-value**
microRNA	–	–	2.28 (1.42–4.14)	0.002	6.28 (1.42–42.4)	0.04	5.86 (1.82–24.6)	0.01	2.18 (1.30–4.19)	0.01
Age	1.04 (0.97–1.11)	0.3	1.02 (0.95–1.09)	0.6	1.03 (0.97–1.11)	0.4	1.02 (0.96–1.10)	0.5	1.03 (0.96–1.10)	0.5
Gender										
Female	Reference	–	Reference	–	Reference	–	Reference	–	Reference	–
Male	6.07 (1.50–30.7)	0.02	4.69 (0.93–31.3)	0.08	5.25 (1.15–30.1)	0.04	6.47 (1.33–40.5)	0.03	7.27 (1.44–49.5)	0.03
Preoperative eGFR	0.93 (0.88–0.99)	0.02	0.91 (0.84–0.97)	0.01	0.93 (0.87–0.98)	0.02	0.91 (0.85–0.97)	0.01	0.91 (0.84–0.97)	0.01
Diabetes										
No	Reference	–	Reference	–	Reference	–	Reference	–	Reference	–
Yes	2.48 (0.62–11.6)	0.2	6.86 (1.26–52.0)	0.04	4.38 (0.99–23.1)	0.06	4.94 (1.04–28.1)	0.05	5.32 (1.08–33.5)	0.05
Predictive accuracy(%)	81		88		83		86		86	

Abbreviations: CI=confidence interval; eGFR=estimated glomerular fraction rate; OR=odds ratio.

## References

[bib1] Arner E, Mejhert N, Kulyte A, Balwierz PJ, Pachkov M, Cormont M, Lorente-Cebrian S, Ehrlund A, Laurencikiene J, Heden P, Dahlman-Wright K, Tanti JF, Hayashizaki Y, Ryden M, Dahlman I, van Nimwegen E, Daub CO, Arner P (2012) Adipose tissue microRNAs as regulators of CCL2 production in human obesity. Diabetes 61(8): 1986–1993. 22688341 10.2337/db11-1508 PMC3402332

[bib2] Benjamini Y, Hochberg Y (1995) Controlling the false discovery rate: a practical and powerful approach to multiple testing. J R Stat Soc Series B Methodol 57(1): 289–300.

[bib3] Cancer Genome Atlas Research N (2013) Comprehensive molecular characterization of clear cell renal cell carcinoma. Nature 499(7456): 43–49. 23792563 10.1038/nature12222 PMC3771322

[bib4] Capitanio U, Larcher A, Terrone C, Antonelli A, Volpe A, Fiori C, Furlan M, Deho F, Minervini A, Serni S, Porpiglia F, Trevisani F, Salonia A, Carini M, Simeone C, Montorsi F, Bertini R (2016) End-stage renal disease after renal surgery in patients with normal preoperative kidney function: balancing surgical strategy and individual disorders at baseline. Eur Urol 70(4): 558–561. 10.1016/j.eururo.2016.03.023 27021797

[bib5] Cascione L, Gasparini P, Lovat F, Carasi S, Pulvirenti A, Ferro A, Alder H, He G, Vecchione A, Croce CM, Shapiro CL, Huebner K (2013) Integrated microRNA and mRNA signatures associated with survival in triple negative breast cancer. PLoS One 8(2): e55910. 23405235 10.1371/journal.pone.0055910 PMC3566108

[bib6] Catto JW, Alcaraz A, Bjartell AS, De Vere White R, Evans CP, Fussel S, Hamdy FC, Kallioniemi O, Mengual L, Schlomm T, Visakorpi T (2011) MicroRNA in prostate, bladder, and kidney cancer: a systematic review. Eur Urol 59(5): 671–681. 21296484 10.1016/j.eururo.2011.01.044

[bib7] Eknoyan G, Lameire N, Eckardt KU, Kasiske BL, Wheeler DC, Abboud OI, Adler S, Agarwal R (2013) KDIGO 2012 Clinical Practice Guideline for the evaluation and management of chronic kidney disease. Kidney Int Suppl 3(1): 5–14.

[bib8] Elvira-Matelot E, Zhou XO, Farman N, Beaurain G, Henrion-Caude A, Hadchouel J, Jeunemaitre X (2010) Regulation of WNK1 expression by miR-192 and aldosterone. J Am Soc Nephrol 21(10): 1724–1731. 20813867 10.1681/ASN.2009111186 PMC3013541

[bib9] Fabbri M, Valeri N, Calin GA (2009) MicroRNAs and genomic variations: from Proteus tricks to Prometheus gift. Carcinogenesis 30(6): 912–917. 19293341 10.1093/carcin/bgp063

[bib10] Fasanaro P, D'Alessandra Y, Magenta A, Pompilio G, Capogrossi MC (2015) microRNAs: promising biomarkers and therapeutic targets of acute myocardial ischemia. Curr Vasc Pharmacol 13(3): 305–315. 23713865 10.2174/15701611113119990011

[bib11] Ferlay J, Steliarova-Foucher E, Lortet-Tieulent J, Rosso S, Coebergh JW, Comber H, Forman D, Bray F (2013) Cancer incidence and mortality patterns in Europe: estimates for 40 countries in 2012. Eur J Cancer 49(6): 1374–1403. 23485231 10.1016/j.ejca.2012.12.027

[bib12] Gastaldi C, Bertero T, Xu N, Bourget-Ponzio I, Lebrigand K, Fourre S, Popa A, Cardot-Leccia N, Meneguzzi G, Sonkoly E, Pivarcsi A, Mari B, Barbry P, Ponzio G, Rezzonico R (2014) miR-193b/365a cluster controls progression of epidermal squamous cell carcinoma. Carcinogenesis 35(5): 1110–1120. 24374827 10.1093/carcin/bgt490

[bib13] Haetscher N, Feuermann Y, Wingert S, Rehage M, Thalheimer FB, Weiser C, Bohnenberger H, Jung K, Schroeder T, Serve H, Oellerich T, Hennighausen L, Rieger MA (2015) STAT5-regulated microRNA-193b controls haematopoietic stem and progenitor cell expansion by modulating cytokine receptor signalling. Nat Commun 6: 8928. 26603207 10.1038/ncomms9928 PMC4674773

[bib14] Hou C, Yang Z, Kang Y, Zhang Z, Fu M, He A, Zhang Z, Liao W (2015) MiR-193b regulates early chondrogenesis by inhibiting the TGF-beta2 signaling pathway. FEBS Lett 589(9): 1040–1047. 25728278 10.1016/j.febslet.2015.02.017

[bib15] Ikeda Y, Tanji E, Makino N, Kawata S, Furukawa T (2012) MicroRNAs associated with mitogen-activated protein kinase in human pancreatic cancer. Mol Cancer Res 10(2): 259–269. 22188669 10.1158/1541-7786.MCR-11-0035

[bib16] Iwamoto N, Vettori S, Maurer B, Brock M, Pachera E, Jungel A, Calcagni M, Gay RE, Whitfield ML, Distler JH, Gay S, Distler O (2016) Downregulation of miR-193b in systemic sclerosis regulates the proliferative vasculopathy by urokinase-type plasminogen activator expression. Ann Rheum Dis 75(1): 303–310. 25384965 10.1136/annrheumdis-2014-205326

[bib17] Karakiewicz PI, Briganti A, Chun FK, Trinh QD, Perrotte P, Ficarra V, Cindolo L, De la Taille A, Tostain J, Mulders PF, Salomon L, Zigeuner R, Prayer-Galetti T, Chautard D, Valeri A, Lechevallier E, Descotes JL, Lang H, Mejean A, Patard JJ (2007) Multi-institutional validation of a new renal cancer-specific survival nomogram. J Clin Oncol 25(11): 1316–1322. 17416852 10.1200/JCO.2006.06.1218

[bib18] Lim LP, Lau NC, Garrett-Engele P, Grimson A, Schelter JM, Castle J, Bartel DP, Linsley PS, Johnson JM (2005) Microarray analysis shows that some microRNAs downregulate large numbers of target mRNAs. Nature 433(7027): 769–773. 15685193 10.1038/nature03315

[bib19] Ljungberg B, Bensalah K, Canfield S, Dabestani S, Hofmann F, Hora M, Kuczyk MA, Lam T, Marconi L, Merseburger AS, Mulders P, Powles T, Staehler M, Volpe A, Bex A (2015) EAU guidelines on renal cell carcinoma: 2014 update. Eur Urol 67(5): 913–924. 25616710 10.1016/j.eururo.2015.01.005

[bib20] Maluf DG, Dumur CI, Suh JL, Scian MJ, King AL, Cathro H, Lee JK, Gehrau RC, Brayman KL, Gallon L, Mas VR (2014) The urine microRNA profile may help monitor post-transplant renal graft function. Kidney Int 85(2): 439–449. 24025639 10.1038/ki.2013.338 PMC3946645

[bib21] Mariusdottir E, Jonsson E, Marteinsson VT, Sigurdsson MI, Gudbjartsson T (2013) Kidney function following partial or radical nephrectomy for renal cell carcinoma: a population-based study. Scand J Urol 47(6): 476–482. 23597159 10.3109/21681805.2013.783624

[bib22] Mendell JT, Olson EN (2012) MicroRNAs in stress signaling and human disease. Cell 148(6): 1172–1187. 22424228 10.1016/j.cell.2012.02.005 PMC3308137

[bib23] Mets E, Van der Meulen J, Van Peer G, Boice M, Mestdagh P, Van de Walle I, Lammens T, Goossens S, De Moerloose B, Benoit Y, Van Roy N, Clappier E, Poppe B, Vandesompele J, Wendel HG, Taghon T, Rondou P, Soulier J, Van Vlierberghe P, Speleman F (2015) MicroRNA-193b-3p acts as a tumor suppressor by targeting the MYB oncogene in T-cell acute lymphoblastic leukemia. Leukemia 29(4): 798–806. 25231743 10.1038/leu.2014.276 PMC4890642

[bib24] Mladinov D, Liu Y, Mattson DL, Liang M (2013) MicroRNAs contribute to the maintenance of cell-type-specific physiological characteristics: miR-192 targets Na+/K+-ATPase beta1. Nucleic Acids Res 41(2): 1273–1283. 23221637 10.1093/nar/gks1228 PMC3553948

[bib25] Nashar K, Egan BM (2014) Relationship between chronic kidney disease and metabolic syndrome: current perspectives. Diabetes Metab Syndr Obes 7: 421–435. 25258547 10.2147/DMSO.S45183 PMC4173754

[bib26] Neviani P, Fabbri M (2015) Exosomic microRNAs in the tumor microenvironment. Front Med (Lausanne) 2: 47. 26258125 10.3389/fmed.2015.00047 PMC4510410

[bib27] Nuovo GJ, Garofalo M, Valeri N, Roulstone V, Volinia S, Cohn DE, Phelps M, Harrington KJ, Vile R, Melcher A, Galanis E, Sehl S, Adair R, Scott K, Rose A, Toogood G, Coffey MC (2012) Reovirus-associated reduction of microRNA-let-7d is related to the increased apoptotic death of cancer cells in clinical samples. Mod Pathol 25(10): 1333–1344. 22699519 10.1038/modpathol.2012.95 PMC4275064

[bib28] Remuzzi G, Grinyo J, Ruggenenti P, Beatini M, Cole EH, Milford EL, Brenner BM (1999) Early experience with dual kidney transplantation in adults using expanded donor criteria. Double Kidney Transplant Group (DKG). J Am Soc Nephrol 10(12): 2591–2598. 10589699 10.1681/ASN.V10122591

[bib29] Siegel RL, Miller KD, Jemal A (2016) Cancer statistics, 2016. CA Cancer J Clin 66(1): 7–30. 26742998 10.3322/caac.21332

[bib30] Thiery JP (2003) Epithelial-mesenchymal transitions in development and pathologies. Curr Opin Cell Biol 15(6): 740–746. 14644200 10.1016/j.ceb.2003.10.006

[bib31] Trionfini P, Benigni A, Remuzzi G (2015) MicroRNAs in kidney physiology and disease. Nat Rev Nephrol 11(1): 23–33. 25385286 10.1038/nrneph.2014.202

[bib32] Valeri N, Braconi C, Gasparini P, Murgia C, Lampis A, Paulus-Hock V, Hart JR, Ueno L, Grivennikov SI, Lovat F, Paone A, Cascione L, Sumani KM, Veronese A, Fabbri M, Carasi S, Alder H, Lanza G, Gafa R, Moyer MP, Ridgway RA, Cordero J, Nuovo GJ, Frankel WL, Rugge M, Fassan M, Groden J, Vogt PK, Karin M, Sansom OJ, Croce CM (2014) MicroRNA-135b promotes cancer progression by acting as a downstream effector of oncogenic pathways in colon cancer. Cancer Cell 25(4): 469–483. 24735923 10.1016/j.ccr.2014.03.006 PMC3995091

[bib33] Vickers AJ, Elkin EB (2006) Decision curve analysis: a novel method for evaluating prediction models. Med Decis Making 26(6): 565–574. 17099194 10.1177/0272989X06295361 PMC2577036

[bib34] Volinia S, Galasso M, Costinean S, Tagliavini L, Gamberoni G, Drusco A, Marchesini J, Mascellani N, Sana ME, Abu Jarour R, Desponts C, Teitell M, Baffa R, Aqeilan R, Iorio MV, Taccioli C, Garzon R, Di Leva G, Fabbri M, Catozzi M, Previati M, Ambs S, Palumbo T, Garofalo M, Veronese A, Bottoni A, Gasparini P, Harris CC, Visone R, Pekarsky Y, de la Chapelle A, Bloomston M, Dillhoff M, Rassenti LZ, Kipps TJ, Huebner K, Pichiorri F, Lenze D, Cairo S, Buendia MA, Pineau P, Dejean A, Zanesi N, Rossi S, Calin GA, Liu CG, Palatini J, Negrini M, Vecchione A, Rosenberg A, Croce CM (2010) Reprogramming of miRNA networks in cancer and leukemia. Genome Res 20(5): 589–599. 20439436 10.1101/gr.098046.109 PMC2860161

[bib35] Westland R, Schreuder MF, van Goudoever JB, Sanna-Cherchi S, van Wijk JA (2014) Clinical implications of the solitary functioning kidney. Clin J Am Soc Nephrol 9(5): 978–986. 24370773 10.2215/CJN.08900813 PMC4011451

[bib36] Wilflingseder J, Regele H, Perco P, Kainz A, Soleiman A, Muhlbacher F, Mayer B, Oberbauer R (2013) miRNA profiling discriminates types of rejection and injury in human renal allografts. Transplantation 95(6): 835–841. 23511211 10.1097/TP.0b013e318280b385 PMC3677100

[bib37] Winter J, Jung S, Keller S, Gregory RI, Diederichs S (2009) Many roads to maturity: microRNA biogenesis pathways and their regulation. Nat Cell Biol 11(3): 228–234. 19255566 10.1038/ncb0309-228

[bib38] Xu P, Zhao Y, Liu M, Wang Y, Wang H, Li YX, Zhu X, Yao Y, Wang H, Qiao J, Ji L, Wang YL (2014) Variations of microRNAs in human placentas and plasma from preeclamptic pregnancy. Hypertension 63(6): 1276–1284. 24664294 10.1161/HYPERTENSIONAHA.113.02647

[bib39] Zhou X, Li Q, Xu J, Zhang X, Zhang H, Xiang Y, Fang C, Wang T, Xia S, Zhang Q, Xing Q, He L, Wang L, Xu M, Zhao X (2016) The aberrantly expressed miR-193b-3p contributes to preeclampsia through regulating transforming growth factor-beta signaling. Sci Rep 6: 19910. 26822621 10.1038/srep19910 PMC4731805

