# Supplementary material for: MicroRNA 193b-3p as a predictive biomarker of chronic kidney disease in patients undergoing radical nephrectomy for renal cell carcinoma
Source: Br J Cancer. 2016 Nov 1;115(11):1343–50. doi: 10.1038/bjc.2016.329 (PMC5129818; doi:10.1038/bjc.2016.329)
Supplement: Supplementary Information [file bjc2016329x1.docx]

**Supplementary Data**: this appendix has been provided by the authors to give readers additional information about their work.

**Title: MicroRNA 193b-3p as a Predictive Biomarker of Chronic Kidney Disease in Patients Undergoing Radical Nephrectomy for Clear-Cell Renal Cancer**

**Authors:** Francesco Trevisani, Michele Ghidini, Alessandro Larcher, Andrea Lampis, Hazel Lote, Paolo Manunta, Maria Teresa Sciarrone Alibrandi, Laura Zagato, Lorena Citterio, Giacomo Dell’Antonio, Cristina Carenzi, Giovambattista Capasso, Massimo Rugge, Paolo Rigotti, Roberto Bertini, Luciano Cascione, Alberto Briganti, Andrea Salonia, Fabio Benigni, Chiara Braconi, Matteo Fassan, Jens Claus Hahne, Francesco Montorsi, Nicola Valeri

**Table of Contents**

Supplementary Table S1 02

Supplementary Table S2 04

Supplementary Table S3 06

Supplementary Table S4 07

Supplementary Table S5 08

Supplementary Figure S1 10

Supplementary Figure S2 11

Supplementary Figure S3 12

Supplementary Figure S4 13

Supplementary Figure S5 14

**Supplementary Tables**

| **Supplementary Table S1.** List of miRs deregulated in the comparison of CKD Versus NKF patients | | | | |
| --- | --- | --- | --- | --- |
| **MicroRNA_ID** | **Average NKF (log)** | **Average CKD (log)** | **Fold Change CKD/NKF (log)** | **p-value** |
| **hsa-miR-1** | **1.37** | **2.97** | **1.60** | **0.01** |
| **hsa-miR-365a-3p** | **1.99** | **3.68** | **1.69** | **0.01** |
| **hsa-miR-30a-5p** | **13.42** | **12.76** | **-0.66** | **0.01** |
| **hsa-miR-139-5p** | **5.40** | **6.91** | **1.51** | **0.02** |
| **hsa-miR-193b-3p** | **1.48** | **2.75** | **1.27** | **0.02** |
| **hsa-miR-4454** | **16.14** | **16.54** | **0.40** | **0.02** |
| **hsa-miR-223-3p** | **5.98** | **7.82** | **1.84** | **0.03** |
| **hsa-miR-363-3p** | **3.02** | **5.14** | **2.12** | **0.04** |
| **hsa-miR-574-3p** | **1.49** | **2.75** | **1.26** | **0.04** |
| **hsa-miR-30d-5p** | **10.93** | **10.60** | **-0.33** | **0.04** |
| **hsa-miR-484** | **0.12** | **0.69** | **0.57** | **0.05** |
| **hsa-miR-1260a** | **8.70** | **9.31** | **0.61** | **0.05** |
| **hsa-miR-299-3p** | **1.34** | **2.44** | **1.10** | **0.05** |
| hsa-miR-151a-5p | 4.47 | 5.77 | 1.30 | 0.06 |
| hsa-miR-720 | 13.40 | 13.83 | 0.43 | 0.06 |
| hsa-miR-150-5p | 7.86 | 8.95 | 1.09 | 0.07 |
| hsa-miR-652-3p | 0.40 | 0.96 | 0.55 | 0.08 |
| hsa-miR-139-3p | 4.06 | 5.17 | 1.11 | 0.08 |
| hsa-miR-4455 | 1.92 | 0.72 | -1.20 | 0.09 |
| hsa-miR-181b-5p+hsa-miR-181d | 5.05 | 6.09 | 1.03 | 0.09 |
| hsa-miR-4286 | 12.17 | 12.90 | 0.73 | 0.09 |
| hsa-miR-216b | 1.15 | 0.39 | -0.76 | 0.11 |
| hsa-miR-374b-5p | 6.12 | 7.26 | 1.13 | 0.11 |
| hsa-miR-320a | 0.60 | 1.33 | 0.73 | 0.11 |
| hsa-miR-196a-5p | 9.31 | 9.65 | 0.34 | 0.11 |
| hsa-miR-1973 | 1.06 | 1.98 | 0.93 | 0.12 |
| hsa-miR-518b | 3.53 | 4.74 | 1.21 | 0.12 |
| hsa-miR-532-3p | 0.36 | 0.80 | 0.44 | 0.13 |
| hsa-miR-30e-5p | 10.08 | 9.29 | -0.79 | 0.13 |
| hsa-miR-146a-5p | 4.59 | 5.59 | 1.00 | 0.13 |
| hsa-miR-27b-3p | 10.97 | 10.77 | -0.21 | 0.14 |
| hsa-miR-203 | 2.24 | 3.40 | 1.17 | 0.14 |
| hsa-let-7d-5p | 10.69 | 10.87 | 0.18 | 0.15 |
| hsa-miR-3147 | 0.55 | 1.00 | 0.46 | 0.15 |
| hsa-miR-199a-3p+hsa-miR-199b-3p | 10.28 | 10.68 | 0.41 | 0.16 |
| hsa-miR-143-3p | 8.25 | 9.92 | 1.67 | 0.16 |
| hsa-miR-9-5p | 4.33 | 5.60 | 1.27 | 0.16 |
| hsa-miR-342-3p | 7.96 | 8.77 | 0.81 | 0.16 |
| hsa-miR-197-3p | 0.82 | 1.54 | 0.72 | 0.17 |
| hsa-miR-423-5p | 8.38 | 7.85 | -0.52 | 0.17 |
| hsa-miR-128 | 1.29 | 1.92 | 0.64 | 0.18 |
| hsa-miR-206 | 0.88 | 1.55 | 0.67 | 0.18 |
| hsa-let-7b-5p | 14.65 | 14.37 | -0.28 | 0.18 |
| hsa-miR-513b | 0.16 | 0.67 | 0.50 | 0.18 |
| In bold miRs with a p-value ≤0.05. NKF= normal kidney function; CKD= chronic kidney disease. | | | | |

| **Supplementary Table S2.** List of miRs deregulated in the comparison of medulla in CKD Versus NKF patients | | | | |
| --- | --- | --- | --- | --- |
| **MicroRNA_ID** | **Average NKF (log)** | **Average CKD (log)** | **Fold Change CKD/NKF (log)** | **p-value** |
| **hsa-miR-193b-3p** | **0.63** | **2.72** | **2.09** | **0.00** |
| **hsa-miR-3147** | **0.22** | **1.38** | **1.16** | **0.01** |
| **hsa-miR-365a-3p** | **1.40** | **3.99** | **2.59** | **0.01** |
| **hsa-miR-299-3p** | **0.73** | **2.60** | **1.87** | **0.01** |
| **hsa-miR-363-3p** | **2.40** | **5.69** | **3.29** | **0.02** |
| **hsa-miR-3151** | **0.18** | **1.61** | **1.43** | **0.03** |
| **hsa-miR-139-5p** | **4.72** | **7.06** | **2.34** | **0.03** |
| **hsa-miR-374b-5p** | **5.25** | **7.54** | **2.29** | **0.03** |
| **hsa-miR-1973** | **0.38** | **2.06** | **1.68** | **0.04** |
| **hsa-miR-652-3p** | **0.24** | **1.15** | **0.91** | **0.05** |
| hsa-miR-19b-3p | 5.03 | 7.49 | 2.46 | 0.06 |
| hsa-miR-30a-5p | 13.48 | 12.66 | -0.82 | 0.06 |
| hsa-miR-200c-3p | 10.70 | 10.02 | -0.68 | 0.06 |
| hsa-miR-1178 | 0.22 | 0.92 | 0.70 | 0.06 |
| hsa-miR-1 | 1.41 | 3.35 | 1.94 | 0.06 |
| hsa-miR-140-5p | 5.62 | 7.72 | 2.10 | 0.06 |
| hsa-miR-151a-5p | 3.81 | 5.89 | 2.08 | 0.06 |
| hsa-miR-195-5p | 5.50 | 7.57 | 2.07 | 0.07 |
| hsa-miR-374a-5p | 6.43 | 8.74 | 2.31 | 0.08 |
| hsa-miR-337-5p | 0.11 | 0.84 | 0.73 | 0.09 |
| hsa-miR-1260a | 8.61 | 9.33 | 0.72 | 0.09 |
| hsa-miR-487b | 0.00 | 0.37 | 0.37 | 0.10 |
| hsa-miR-215 | 0.00 | 0.32 | 0.32 | 0.10 |
| hsa-miR-4455 | 2.66 | 0.89 | -1.77 | 0.10 |
| hsa-miR-206 | 0.94 | 2.35 | 1.41 | 0.10 |
| hsa-miR-181b-5p+hsa-miR-181d | 5.11 | 6.44 | 1.33 | 0.11 |
| hsa-miR-204-5p | 11.25 | 11.92 | 0.67 | 0.11 |
| hsa-miR-379-5p | 0.00 | 0.35 | 0.35 | 0.11 |
| hsa-miR-182-5p | 1.31 | 0.20 | -1.11 | 0.11 |
| hsa-miR-146a-5p | 4.06 | 5.65 | 1.59 | 0.11 |
| hsa-miR-489 | 4.74 | 6.03 | 1.29 | 0.12 |
| hsa-miR-342-3p | 6.95 | 8.67 | 1.72 | 0.12 |
| hsa-miR-2682-5p | 2.01 | 0.56 | -1.45 | 0.13 |
| hsa-miR-455-3p | 1.02 | 2.16 | 1.14 | 0.13 |
| hsa-miR-532-3p | 0.26 | 0.95 | 0.69 | 0.13 |
| hsa-miR-331-3p | 1.45 | 2.68 | 1.23 | 0.13 |
| hsa-miR-130a-3p | 10.34 | 10.63 | 0.29 | 0.13 |
| hsa-miR-660-5p | 6.27 | 8.22 | 1.95 | 0.14 |
| hsa-miR-4454 | 16.12 | 16.54 | 0.42 | 0.14 |
| hsa-let-7d-5p | 10.71 | 10.98 | 0.27 | 0.15 |
| hsa-miR-139-3p | 4.06 | 5.57 | 1.51 | 0.15 |
| hsa-miR-15b-5p | 6.56 | 8.47 | 1.91 | 0.15 |
| hsa-miR-484 | 0.10 | 0.86 | 0.76 | 0.15 |
| hsa-miR-24-3p | 5.99 | 7.70 | 1.71 | 0.15 |
| hsa-miR-135a-5p | 3.97 | 5.90 | 1.93 | 0.16 |
| hsa-miR-4286 | 11.84 | 12.75 | 0.91 | 0.16 |
| hsa-miR-196a-5p | 9.34 | 9.80 | 0.46 | 0.16 |
| hsa-miR-324-5p | 5.01 | 6.36 | 1.35 | 0.17 |
| hsa-miR-720 | 13.34 | 13.79 | 0.45 | 0.17 |
| hsa-miR-33a-5p | 0.34 | 0.89 | 0.55 | 0.17 |
| hsa-miR-28-5p | 4.97 | 6.57 | 1.60 | 0.17 |
| hsa-miR-186-5p | 7.19 | 6.58 | -0.61 | 0.17 |
| hsa-miR-150-5p | 7.81 | 9.16 | 1.35 | 0.17 |
| hsa-miR-514b-5p | 0.00 | 0.61 | 0.61 | 0.17 |
| hsa-miR-193a-3p | 0.27 | 0.79 | 0.52 | 0.18 |
| hsa-miR-223-3p | 5.81 | 7.71 | 1.90 | 0.18 |
| hsa-miR-143-3p | 7.64 | 10.09 | 2.45 | 0.18 |
| hsa-miR-183-5p | 1.38 | 0.39 | -0.99 | 0.19 |
| has-let-7a-5p | 14.52 | 14.95 | 0.43 | 0.19 |
| In bold miRs with a p-value ≤0.05. NKF= normal kidney function; CKD= chronic kidney disease. | | | | |

| **Supplementary Table S3.** List of miRs deregulated in the comparison of cortex in CKD Versus NKF patients | | | | |
| --- | --- | --- | --- | --- |
| **MicroRNA_ID** | **Average NKF (log)** | **Average CKD (log)** | **Fold Change CKD/NKF (log)** | **p-value** |
| **hsa-miR-216b** | **2.21** | **0.54** | **-1.67** | **0.04** |
| **hsa-miR-31-5p** | **0.14** | **1.51** | **1.37** | **0.04** |
| **hsa-miR-320a** | **0.26** | **1.22** | **0.96** | **0.05** |
| hsa-miR-197-3p | 0.40 | 1.27 | 0.87 | 0.07 |
| hsa-miR-192-5p | 8.98 | 7.26 | -1.72 | 0.07 |
| hsa-miR-574-3p | 0.91 | 2.24 | 1.33 | 0.07 |
| hsa-miR-194-5p | 10.43 | 9.44 | -0.99 | 0.07 |
| hsa-miR-4454 | 16.16 | 16.54 | 0.38 | 0.08 |
| hsa-miR-1 | 1.33 | 2.58 | 1.25 | 0.08 |
| hsa-miR-223-3p | 6.13 | 7.93 | 1.80 | 0.08 |
| hsa-miR-378a-3p+hsa-miR-378i | 8.53 | 7.46 | -1.07 | 0.10 |
| hsa-miR-2682-5p | 0.12 | 1.31 | 1.19 | 0.12 |
| hsa-miR-30d-5p | 10.93 | 10.62 | -0.31 | 0.13 |
| hsa-miR-30a-5p | 13.37 | 12.86 | -0.51 | 0.14 |
| hsa-miR-484 | 0.13 | 0.52 | 0.39 | 0.16 |
| hsa-miR-92a-3p | 3.70 | 5.01 | 1.31 | 0.17 |
| hsa-miR-125b-5p | 12.64 | 12.96 | 0.32 | 0.17 |
| hsa-miR-183-5p | 0.11 | 0.82 | 0.71 | 0.19 |
| In bold miRs with a p-value ≤0.05. NKF= normal kidney function; CKD= chronic kidney disease. | | | | |

| **Supplementary Table S4.** MicroRNA expression in 71 patients treated with radical nephrectomy for kidney cancer according to development of postoperative chronic kidney disease. | | | |
| --- | --- | --- | --- |
| **Variable** | **NKF (n=33)** | **CKD (n=38)** | **p-value** |
| **miR-193b-3p** |  |  | 0.001 |
| Median | 1.54 | 2.83 |  |
| IQR | 0.86-2.08 | 1.53-4.44 |  |
| **miR-363b** |  |  | 0.01 |
| Median | 0.23 | 0.47 |  |
| IQR | 0.12-0.5 | 0.18-0.89 |  |
| **miR-139b** |  |  | 0.003 |
| Median | 0.48 | 0.84 |  |
| IQR | 0.18-0.6 | 0.49-1.38 |  |
| **miR-365a** |  |  | 0.02 |
| Median | 0.61 | 1.14 |  |
| IQR | 0.17-1.43 | 0.44-2.97 |  |
| Data presented as median or interquartile range [IQR]. NKF= normal kidney function; CKD= chronic kidney disease. | | | |

| **Supplementary Table S5.** MicroRNA expression comparison in renal parenchyma matching RCC (n=24 patients) Versus healthy tissues of kidney donors (n=12) | | | | |
| --- | --- | --- | --- | --- |
| **microRNA_ID** | **Average Healthy Kidney Donors (log)** | **Average "Normal" tissue matching RCC**  **(log)** | **Fold Change “Normal”/ Donors**  **(log)** | **p-value** |
| hsa-let-7g-5p | 13.42 | 12.79 | -0.64 | 0.0480 |
| hsa-miR-26a-5p | 12.59 | 11.86 | -0.74 | 0.0476 |
| hsa-miR-29a-3p | 9.84 | 10.49 | 0.65 | 0.0462 |
| hsa-miR-23b-3p | 11.69 | 11.19 | -0.50 | 0.0324 |
| hsa-let-7f-5p | 11.95 | 9.96 | -1.99 | 0.0298 |
| hsa-miR-30a-5p | 12.28 | 13.08 | 0.81 | 0.0253 |
| hsa-miR-362-5p | 7.57 | 8.40 | 0.82 | 0.0220 |
| hsa-miR-146a-5p | 7.17 | 5.10 | -2.08 | 0.0204 |
| hsa-miR-200b-3p | 11.72 | 12.27 | 0.55 | 0.0191 |
| hsa-miR-95 | 6.05 | 4.01 | -2.04 | 0.0173 |
| hsa-miR-30b-5p | 12.10 | 10.48 | -1.62 | 0.0157 |
| hsa-miR-1180 | 3.14 | 5.32 | 2.18 | 0.0141 |
| hsa-miR-181c-5p | 0.91 | 3.23 | 2.32 | 0.0102 |
| hsa-miR-222-3p | 8.47 | 9.21 | 0.74 | 0.0102 |
| hsa-miR-125a-5p | 10.68 | 9.70 | -0.98 | 0.0098 |
| hsa-miR-130a-3p | 9.93 | 10.44 | 0.51 | 0.0070 |
| hsa-miR-345-5p | 0.70 | 0.12 | -0.57 | 0.0070 |
| hsa-miR-720 | 14.48 | 13.62 | -0.86 | 0.0049 |
| hsa-miR-181a-5p | 9.75 | 10.76 | 1.01 | 0.0044 |
| hsa-miR-484 | 1.77 | 0.41 | -1.36 | 0.0038 |
| hsa-miR-1972 | 0.00 | 3.65 | 3.65 | 0.0033 |
| hsa-miR-1178 | 1.98 | 0.56 | -1.42 | 0.0026 |
| hsa-miR-331-3p | 4.61 | 2.39 | -2.22 | 0.0021 |
| hsa-miR-29b-3p | 10.89 | 11.89 | 1.00 | 0.0015 |
| hsa-let-7d-5p | 11.39 | 10.78 | -0.60 | 0.0012 |
| hsa-miR-507 | 2.13 | 0.37 | -1.75 | 0.0012 |
| hsa-miR-128 | 3.75 | 1.61 | -2.14 | 0.0012 |
| hsa-miR-144-3p | 0.38 | 4.54 | 4.16 | 0.0011 |
| hsa-miR-483-3p | 4.10 | 1.64 | -2.45 | 0.0009 |
| hsa-let-7b-5p | 13.56 | 14.51 | 0.95 | 0.0007 |
| hsa-miR-142-3p | 5.96 | 8.08 | 2.12 | 0.0006 |
| hsa-miR-574-3p | 5.02 | 2.13 | -2.88 | 0.0005 |
| hsa-miR-30c-5p | 11.35 | 10.11 | -1.23 | 0.0002 |
| hsa-miR-99a-5p | 11.07 | 11.84 | 0.78 | 0.0002 |
| hsa-miR-532-3p | 2.52 | 0.59 | -1.93 | 0.0001 |
| hsa-miR-574-5p | 3.13 | 6.16 | 3.02 | 0.0001 |
| hsa-miR-494 | 5.73 | 9.09 | 3.37 | <0.0001 |
| hsa-miR-107 | 10.61 | 9.23 | -1.38 | <0.0001 |
| hsa-miR-365a-3p | 6.79 | 2.85 | -3.94 | <0.0001 |
| hsa-miR-215 | 3.67 | 0.94 | -2.73 | <0.0001 |
| hsa-miR-514a-3p | 7.24 | 2.22 | -5.02 | <0.0001 |
| hsa-miR-204-5p | 13.74 | 12.05 | -1.69 | <0.0001 |
| hsa-miR-1260a | 11.02 | 9.02 | -2.00 | <0.0001 |
| hsa-miR-542-5p | 3.49 | 0.64 | -2.86 | <0.0001 |
| hsa-miR-3147 | 3.08 | 0.78 | -2.30 | <0.0001 |
| hsa-miR-197-3p | 5.13 | 1.19 | -3.94 | <0.0001 |
| hsa-miR-455-3p | 5.76 | 1.70 | -4.06 | <0.0001 |
| hsa-miR-509-3p | 5.52 | 0.84 | -4.68 | <0.0001 |
| hsa-miR-506-3p | 3.62 | 0.16 | -3.46 | <0.0001 |
| hsa-miR-508-3p | 6.43 | 0.78 | -5.65 | <0.0001 |
| hsa-miR-513c-5p | 3.12 | 0.05 | -3.07 | <0.0001 |
| hsa-miR-451a | 3.58 | 12.16 | 8.58 | <0.0001 |
| hsa-miR-514b-5p | 6.39 | 0.46 | -5.94 | <0.0001 |
| hsa-miR-513b | 6.99 | 0.42 | -6.57 | <0.0001 |
| RCC= Clear-Cell Renal Carcinoma | | | | |

**Supplementary FIGURES**

**
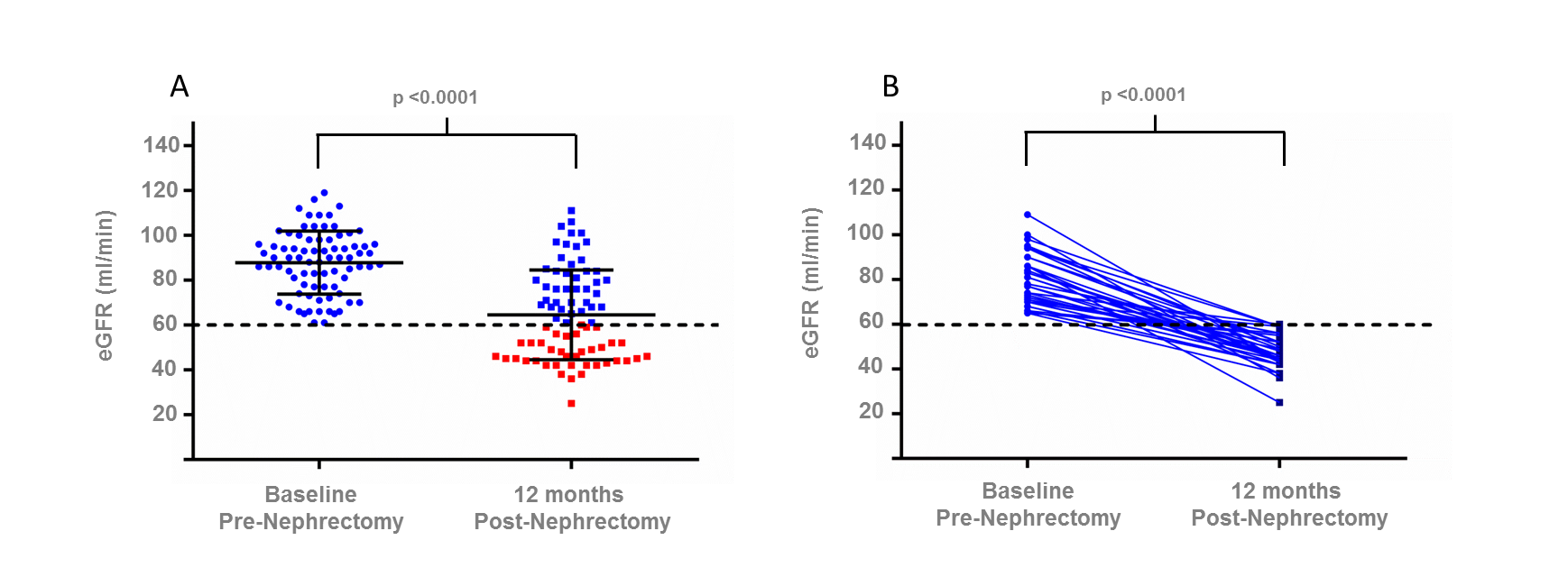
**

**Figure S1.** Estimated Glomerular Fraction Rate (eGFR) reduction measured 12 months post nephrectomy in the study population. (A) Thirty-eight patients experienced a reduction in eGFR below 60 mil/min 1 year post-operatively. Blue dots= normal eGFR; Red dots= abnormal eGFR. (B) Blue lines identify eGFR reduction in patients developing CKD.


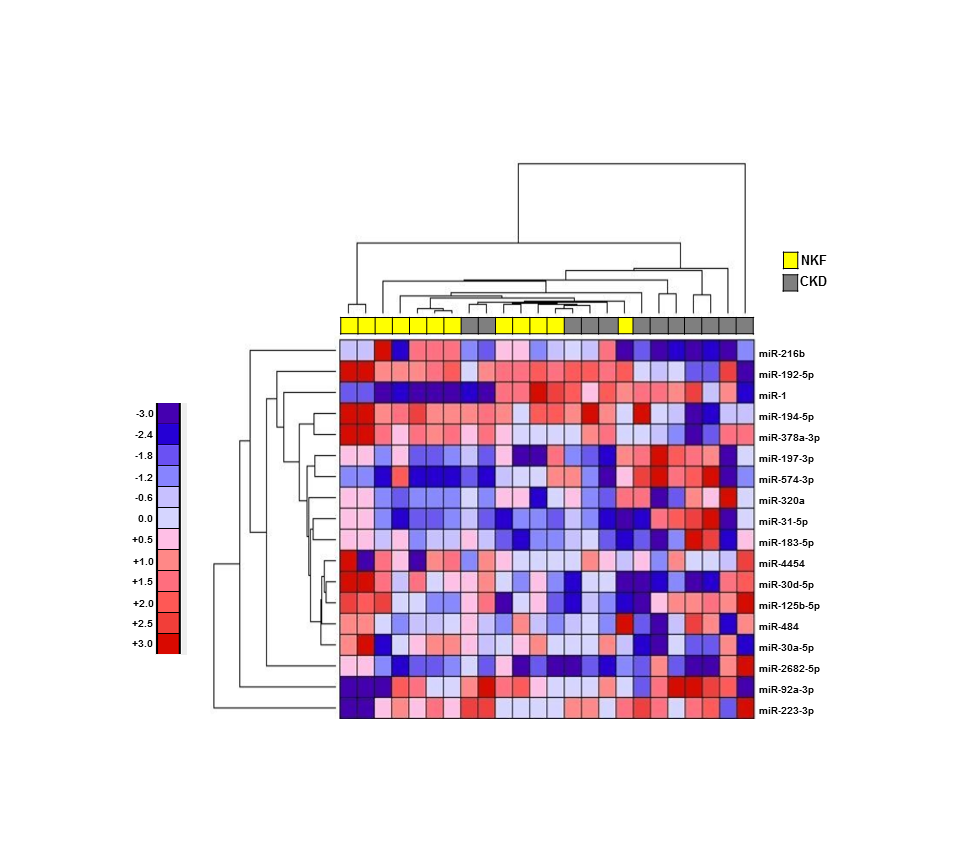


**Supplementary Figure S2.** Heatmap showing miR expression in cortex of patients with normal kidney function (NKF) compared to patients developing Chronic Kidney Disease (CKD) 12 months post nephrectomy for RCC. miRs with p value ≤0.1 are shown.


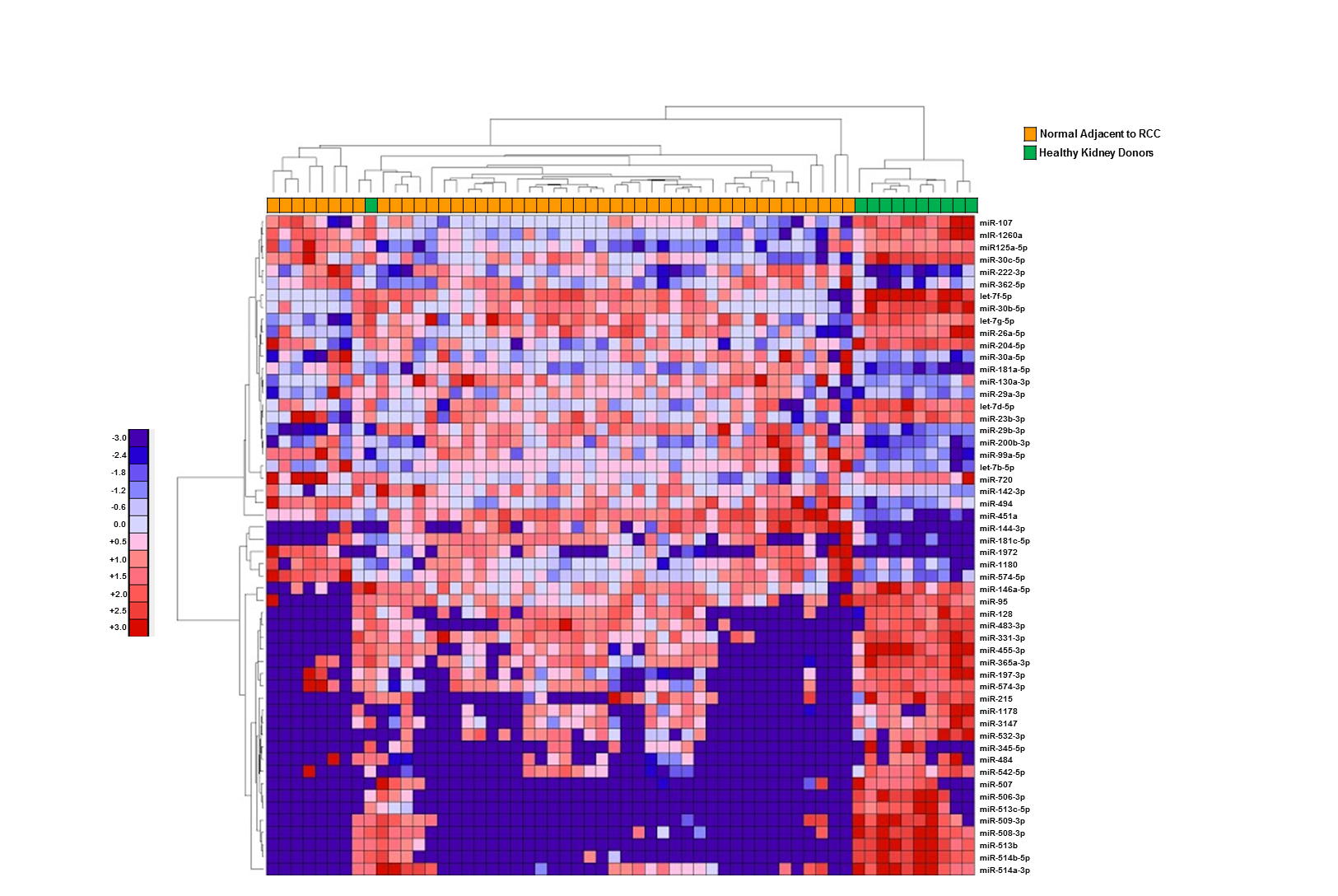


**Supplementary Figure S3.** Heatmap showing miR expression in cortex of healthy kidney donors (n=11) compared to parenchyma adjacent to RCC (n=23 medulla + 24 cortex). MiRs with an adjusted p-value ≤0.05 are shown.


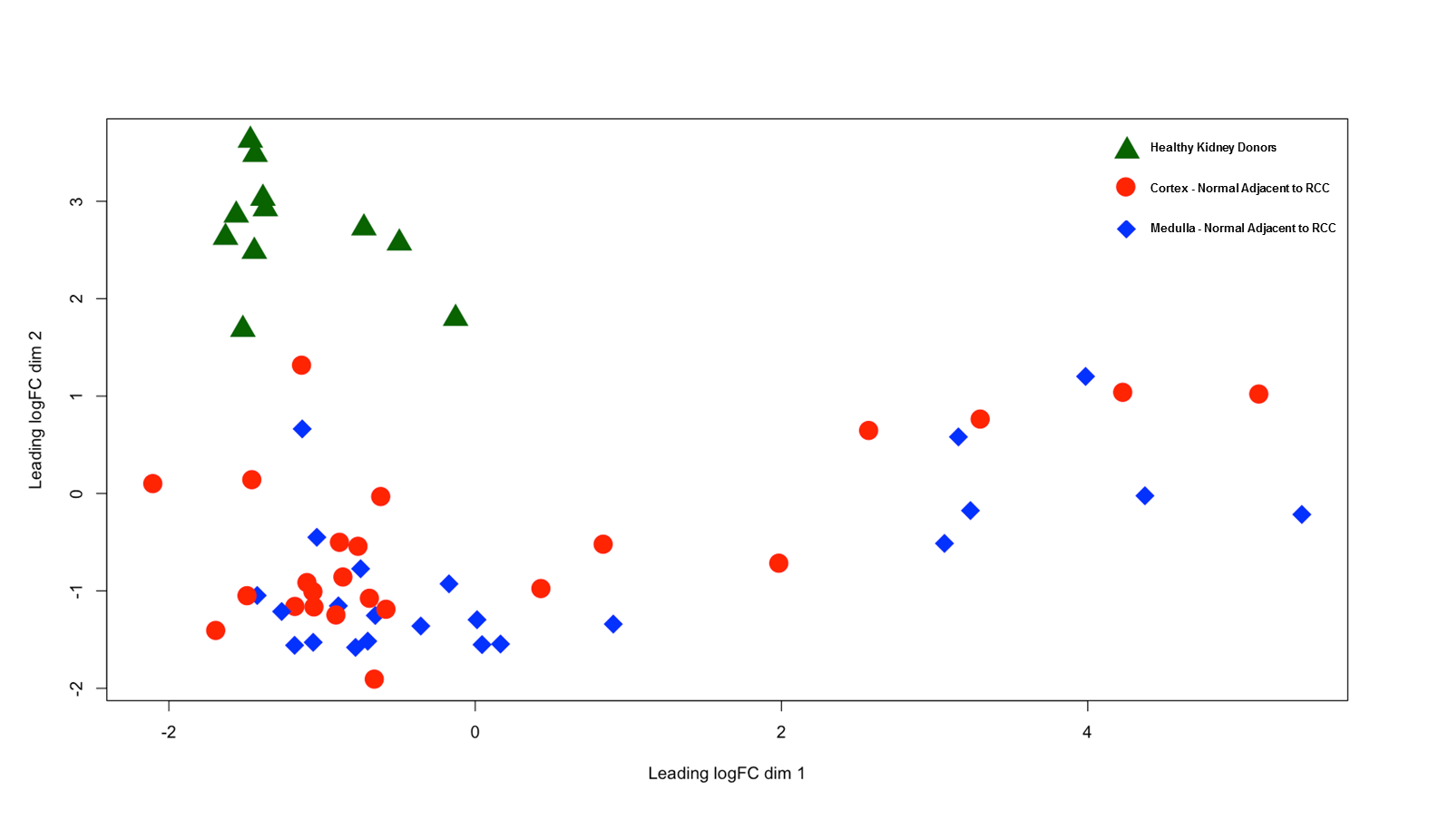


**Supplementary Figure S4.** Multidimensional scaling plot of miR expression data on healthy kidney donors (n=11), medulla (n=23) and cortex (n=24) of patients receiving RN for RCC following quantile normalisation and background correction. It shows the relationship among samples; distances represent leading log2-fold changes between them. Green triangles represent healthy kidney donors, blue diamonds and red bullets represent medulla and cortex parenchyma matching RCC in the training cohort.

**
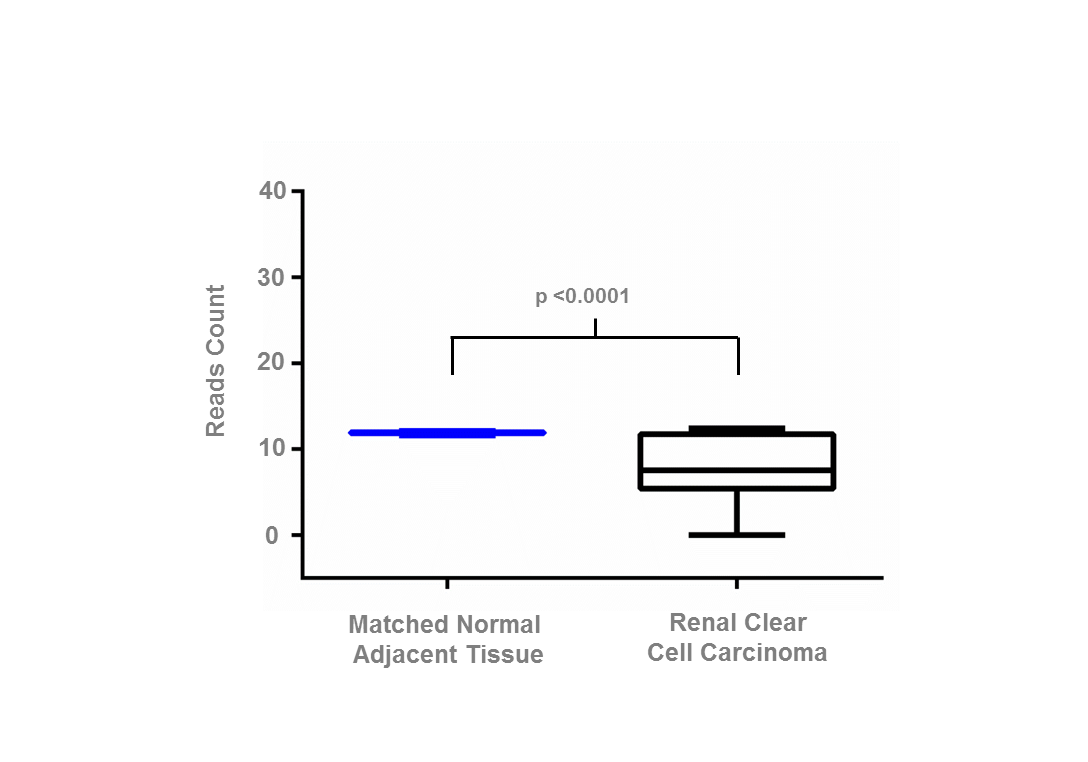
**

**Supplementary Figure S5.** Plot showing miR-193b-3p expression (miR-seq reads) in 71 patients with RCC and matched normal tissue included in The Cancer Genome Atlas dataset.
